# Supplementary material for: CriTER-A: A Novel Temperature-Dependent Noncoding RNA Switch in the Telomeric Transcriptome of Chironomus riparius
Source: Int J Mol Sci. 2021 Sep 24;22(19):10310. doi: 10.3390/ijms221910310 (PMC8508857; doi:10.3390/ijms221910310)
Supplement: Supplementary file 1 [file ijms-22-10310-s001.zip › ijms-1374470-supplementary.pdf]

Supplementary Material for

CriTER-A: a novel temperature-dependent noncoding RNA switch in the telomeric transcriptome of *Chironomus riparius*

Cristina Romero-López [1,*], Alfredo Berzal-Herranz [1], José Luis Martínez-Guitarte[2] and Mercedes de la Fuente [3,*]

[1] Instituto de Parasitología y Biomedicina López-Neyra (IPBLN-CSIC), Av. Conocimiento 17, Armilla, 18016 Granada, Spain; CRL, cristina_romero@ipb.csic.es; ABH, aberzalh@ipb.csic.es.

[2] Grupo de Biología y Toxicología Ambiental. Facultad de Ciencias. Universidad Nacional de Educación a Distancia (UNED), jlmartinez@ccia.uned.es

[3] Departamento de Ciencias y Técnicas Fisicoquímicas, Universidad Nacional de Educación a Distancia (UNED), Avda. de Esparta, s/n, 28230, Las Rozas, Spain, mfuente@ccia.uned.es

* Correspondence: CRL, cristina_romero@ipb.csic.es; MF, mfuente@ccia.uned.es

Content index

This PDF file includes:

- **CriTER-A. Secondary Structure Prediction.** Includes **Figures S1 to S3**.
- **CriTER-B. Secondary Structure Prediction.** Includes **Figures S4 to S7**.

CriTER-A. Secondary Structure Prediction

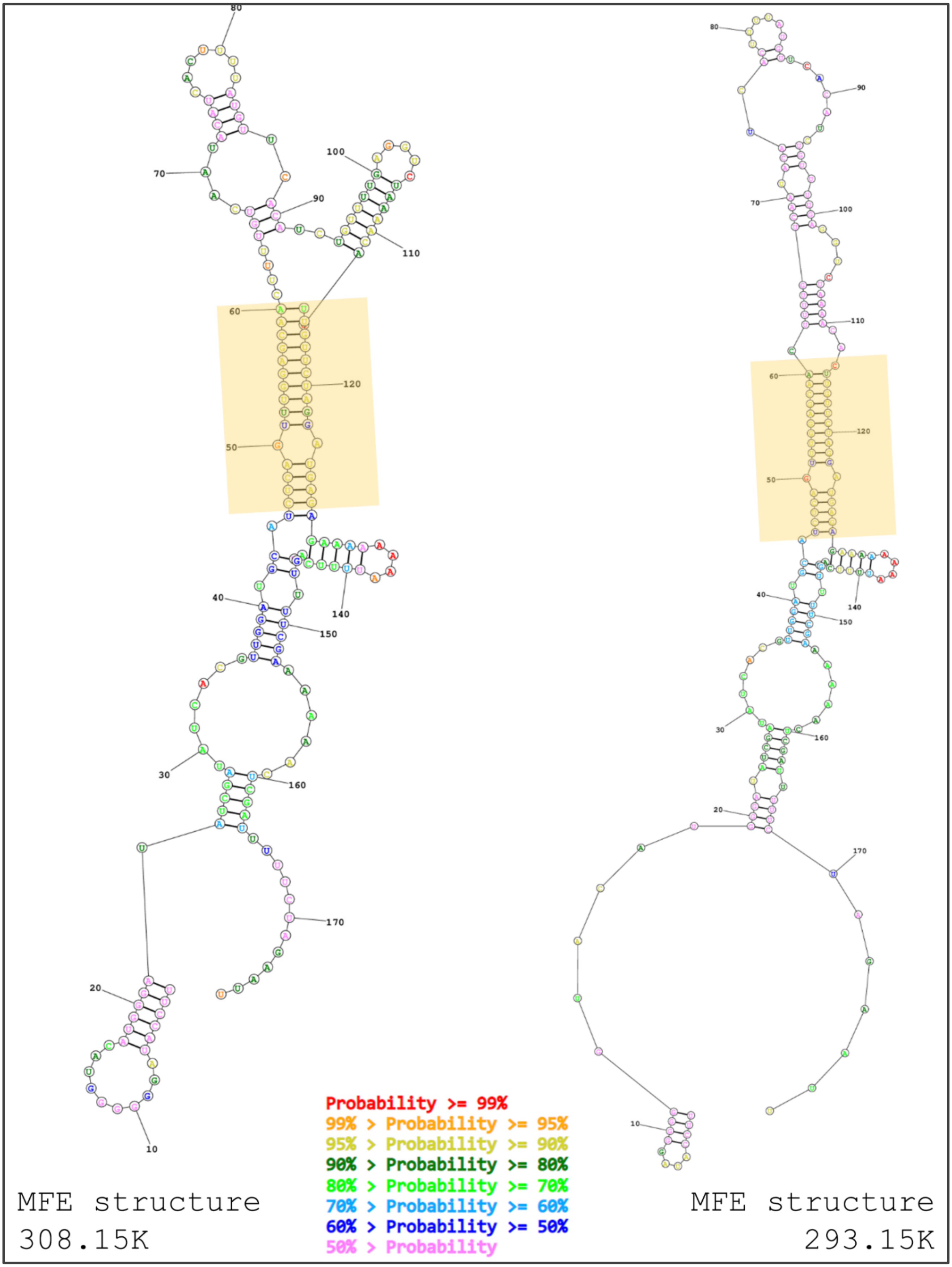

Figure S1. The minimun free energy (MFE) structure predicted for CriTER-A at 308,15 K (35°C) and 293,15 K (20°C). Calculations were performed with RNAStructure 6.3 using default parameters. The region involving high base pairing probabilities is highlighted (shadowed).

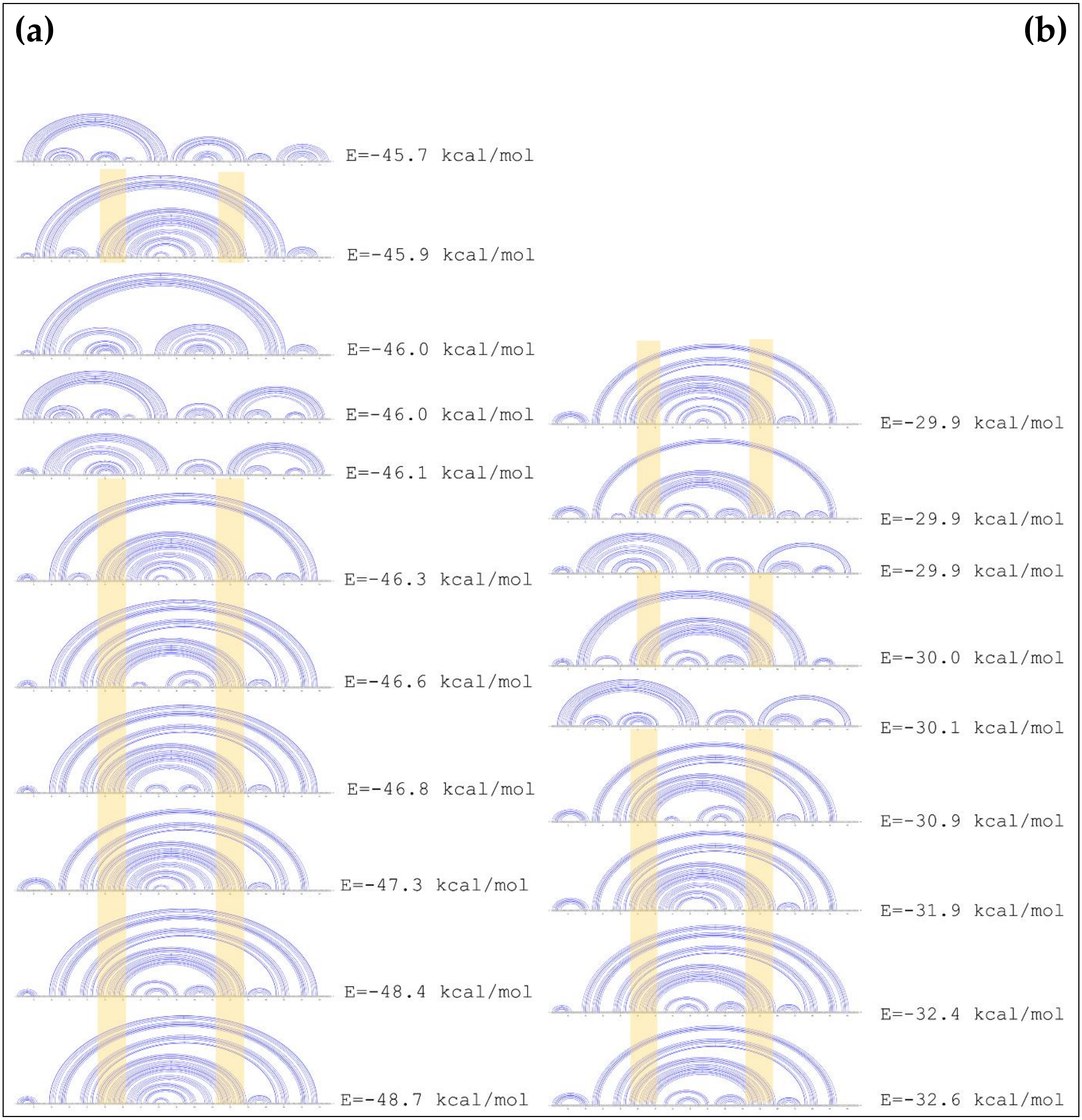

Figure S2. Arc diagram of predicted MFE and suboptimal structures in a energy range of 3 kcal/mol for CriTER-A. Calculations were performed with RNAStructure 6.3 using default parameters at (a) 293,15 K (20°C) and (**b**) 305,15 K (35°C). The region involving high base pairing probabilities is highlighted (shadowed).

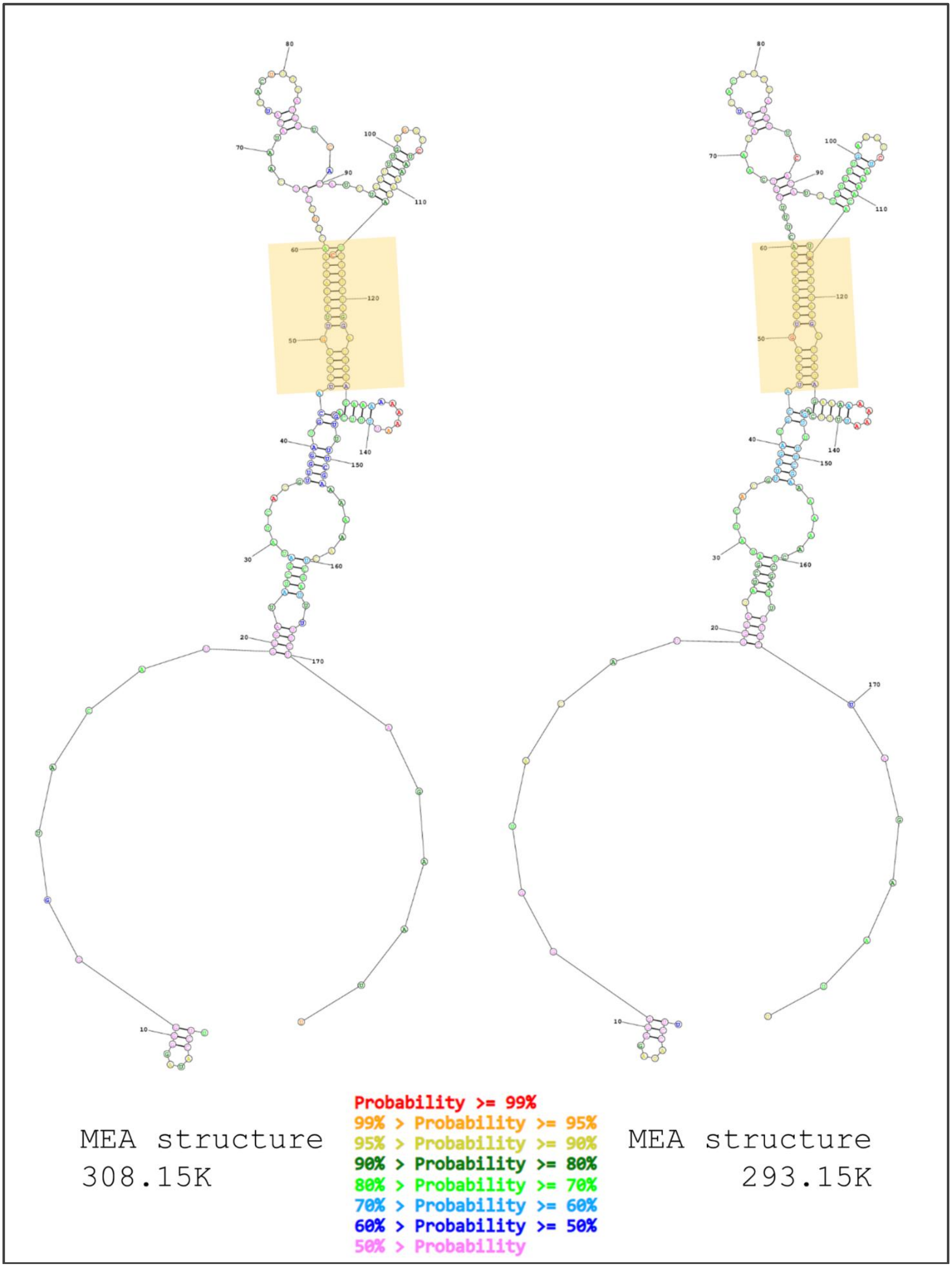

Figure S3. The maximum expected accuracy (MEA) structure predicted for CriTER-A at 308,15 K (35°C) and 293,15 K (20°C). Calculations were performed with RNAStructure 6.3 using default parameters. The region involving high base pairing probabilities is highlighted (shadowed).

CriTER-B. Secondary Structure Prediction

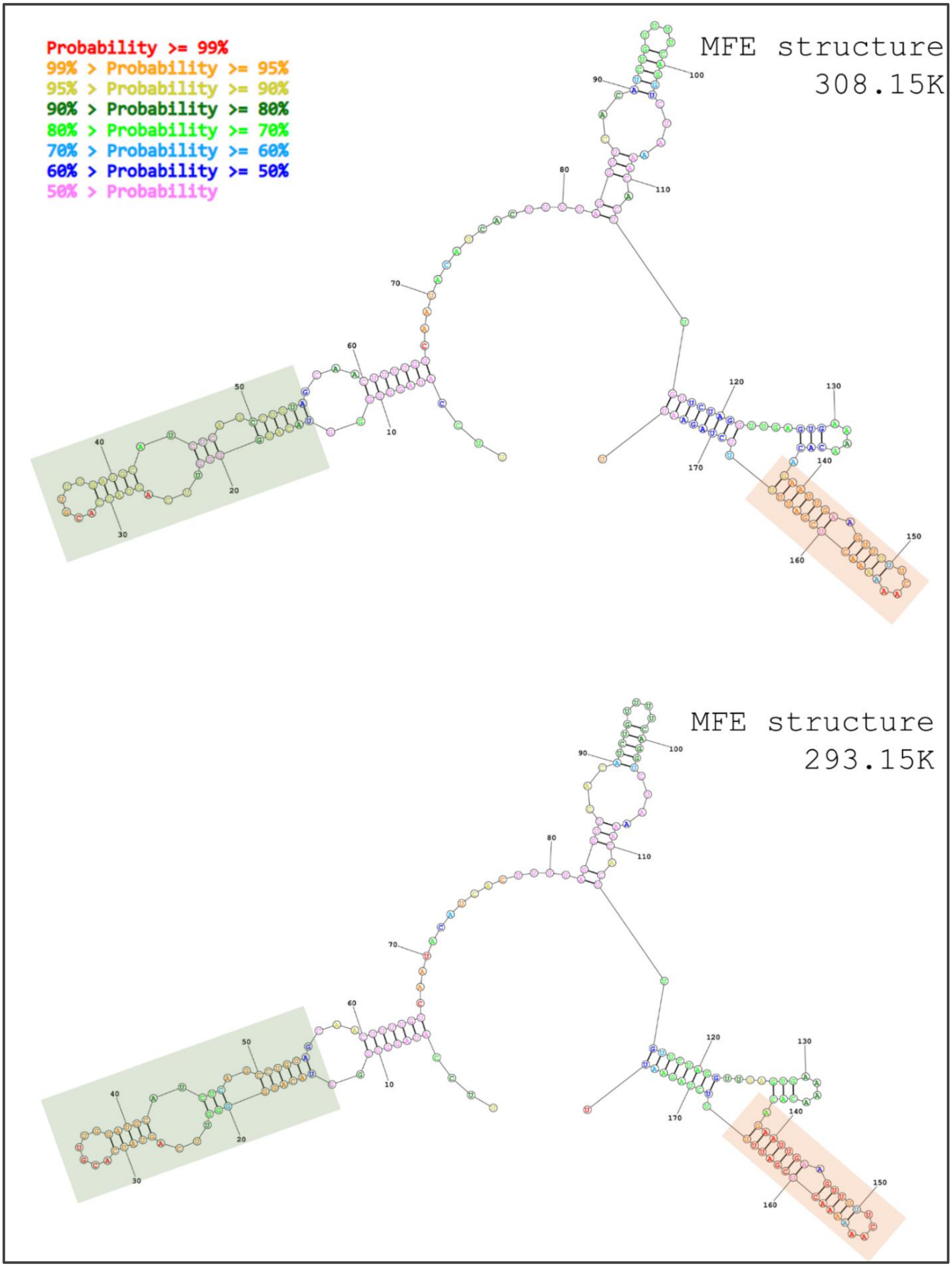

Figure S4. The minimun free energy (MFE) structure predicted for CriTER-B at 308,15 K (35°C) and 293,15 K (20°C). Calculations were performed with RNAStructure 6.3 using default parameters. The two stem-loops involving high base pairing probabilities are highlighted (shadowed).

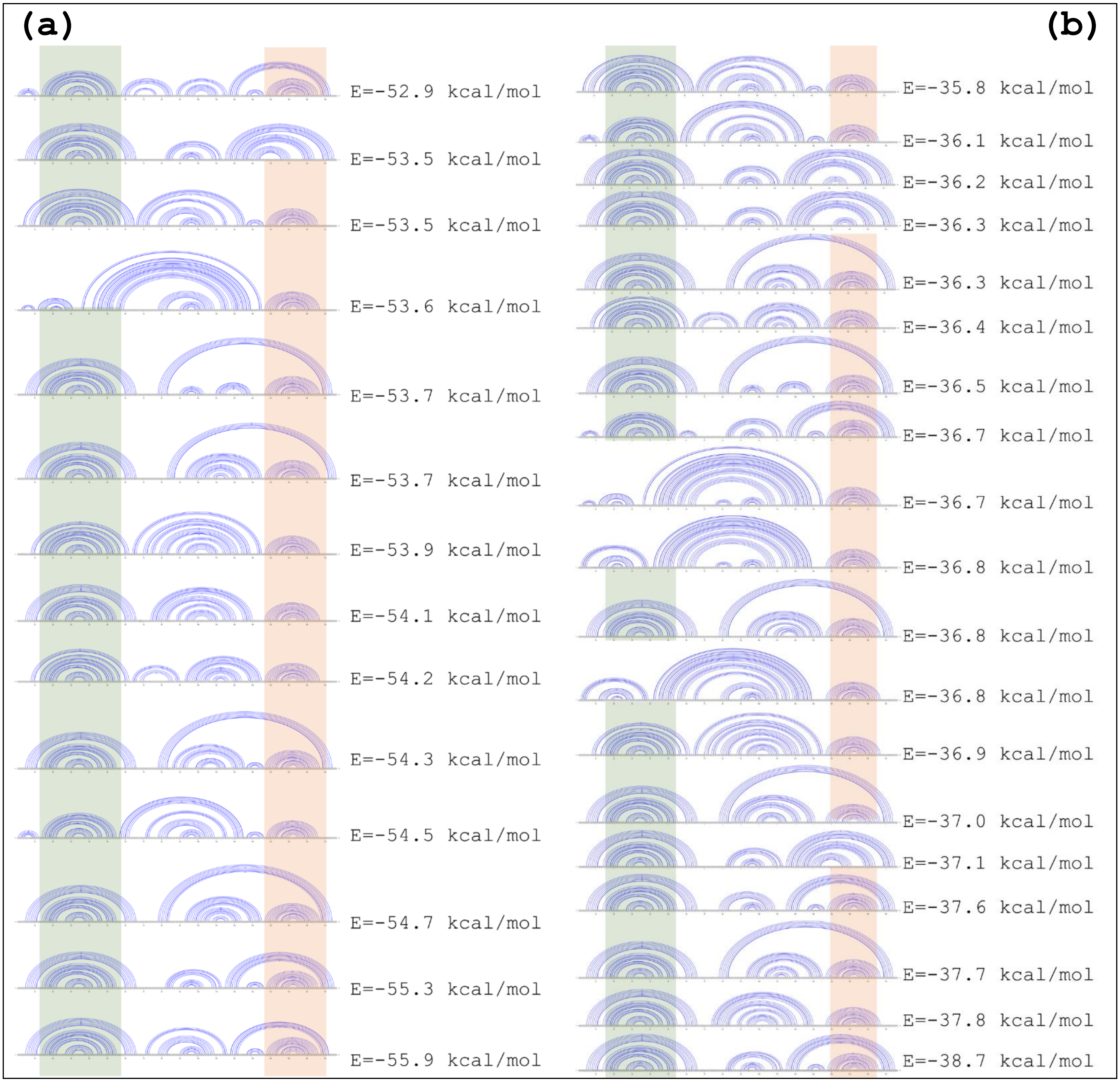

Figure S5. Arc diagram of predicted MFE and suboptimal structures in an energy range of 3 kcal/mol for CriTER-B. Calculations were performed with RNAStructure 6.3 using default parameters at (a) 293,15 K (20°C) and (b) 305,15 K (35°C). The two stem-loops involving high base pairing probabilities are highlighted (shadowed).

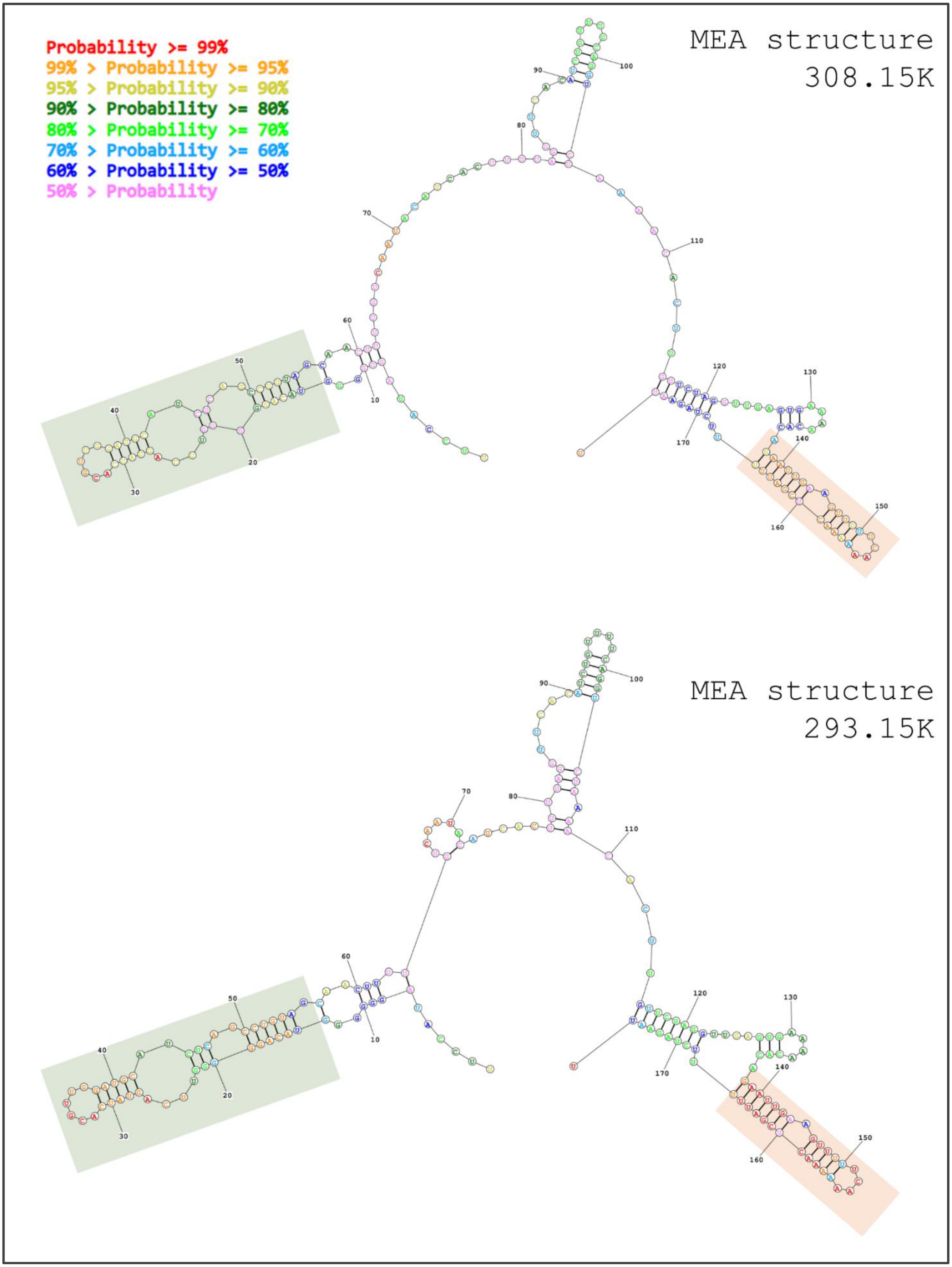

Figure S6. The maximum expected accuracy (MEA) structure predicted for CriTER-B at 308,15 K (35°C) and 293,15 K (20°C). Calculations were performed with RNAStructure 6.3 using default parameters. The two stem-loops involving high base pairing probabilities are highlighted (shadowed).

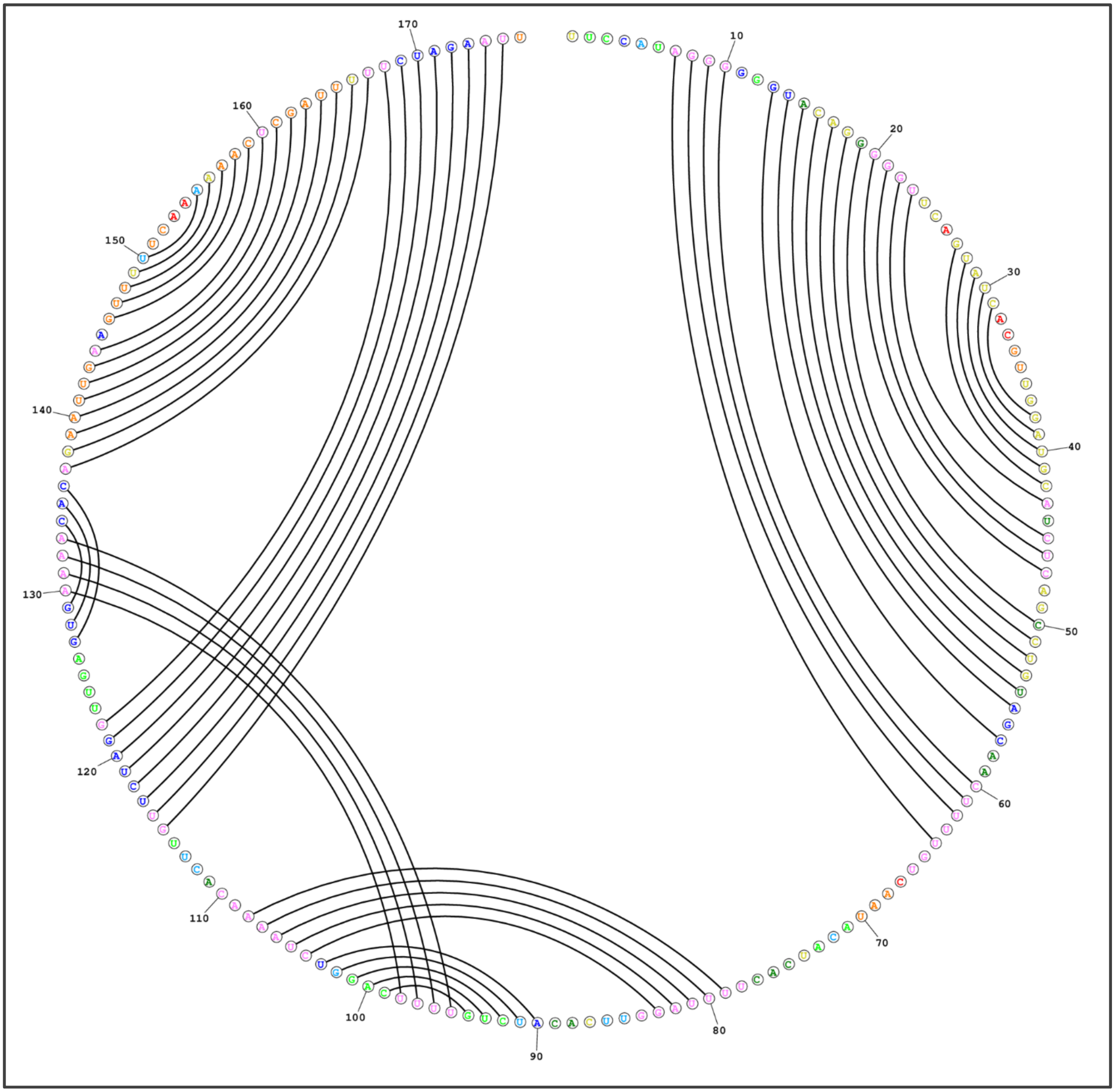

Figure S7. Secondary structure circular representation of probable base pairs of CriTER-B, including putative pseudoknots. Calculations were performed with RNAStructure 6.3 using default parameters.
